# Tracheostomy Complication in a Burn Patient

**Published:** 2014-01-09

**Authors:** Jonathan S. Lam, Leigh A. Price, Stephen M. Milner

**Affiliations:** Department of Plastic and Reconstructive Surgery, Johns Hopkins University School of Medicine, Baltimore, MD

**Keywords:** tracheocutaneous fistula, persistent tracheostomy stoma, airway, trachea, burn

## DESCRIPTION

**Figure 1:**
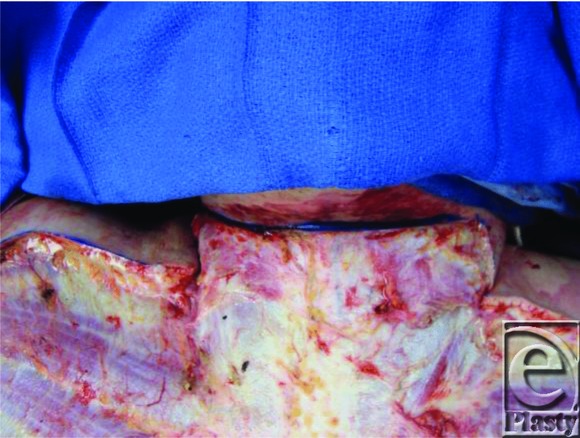
Fascial excision of neck.

**Figure 2:**
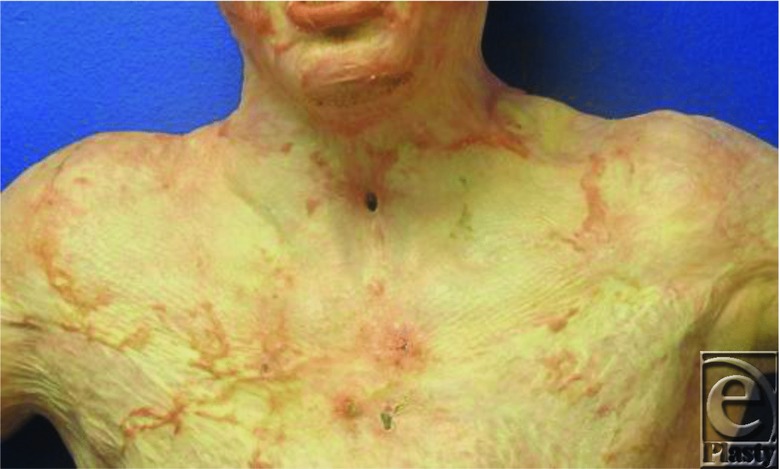
Midline neck defect following tracheostomy decannulation after one year.

A 20-year-old man presented with 90% total body surface area third-degree burns from a gasoline fire in his basement. He underwent extensive fascial excisions of his burn wounds, including his neck and upper chest (Fig 1). All wounds were resurfaced with cultured epithelial autograft. The patient's tracheostomy tube was decannulated after 1 year; however, his stoma failed to close spontaneously (Fig 2). The patient voiced concerns about the cosmetic deformity, skin irritation, and mucous secretions.

## QUESTIONS

1. **What is the cause and pathophysiology of the condition?**
2. **What is the morbidity associated with them?**
3. **What are the management options?**
4. **How does the surgical treatment differ in the burn patient?**

## DISCUSSION

The patient has a persistent tracheocutaneous fistula (TCF) which is a well-recognized complication of long-term tracheostomies. Removal of the tracheostomy tube usually results in rapid narrowing of the stoma, followed by spontaneous closure of the overlying skin; however, the tract may be more prone to fistula formation depending on the duration of cannulation. Longer periods of cannulation predispose the tract to epithelialization, reducing the ability to close. Kulber and Passy^1^ reported that fistulae do not form when the cannulation period is less than 16 weeks; however, the likelihood increases to 70% when the cannulation period exceeds 16 weeks.

The morbidity of persistent TCFs has both functional and cosmetic aspects. A TCF can cause difficulty in phonation and swallowing, skin irritation, intolerance to submersion due to aspiration risk, and ineffective clearance of secretions. In addition, unsightly deformities can lead to self-stigmatization.^2^^,^^3^

The best method of management of TCFs is prevention. The need for tracheostomy must be continuously evaluated, and they should be removed expeditiously when no longer needed. However, burned patients may require long-term tracheostomies because of the multitude of surgical procedures and intensive care. Other management options include curettage and application of silver nitrate. In addition, electrocautery of the epithelial tract has been used to remove granulation tissue and its bioburden while promoting healthy tissue for wound contracture and ultimate healing. Several successful surgical techniques have been described including primary closure, fistulectomy with primary closure, and fistulectomy with closure by secondary intention. Bishop et al^4^ described a 2-layer closure without fistulectomy composed of sternohyoid muscle flaps and a transverse skin closure. The important principle of his repair was to provide separation of the 2 suture lines (skin and trachea) by intervening muscle. A 3-layer closure was described by Schroeder et al,^3^ in which the skin edges and fistula tract were excised down to but not into the trachea while leaving a distal 4-mm cuff of the fistula contiguous with the trachea. The cuff, being the first layer, was closed transversely to provide an airtight seal. A drain was subsequently placed anterior to this layer to prevent development of subcutaneous emphysema and pneumomediastinum. Sequentially, the strap muscles and skin were closed, completing the repair. A similar technique was described earlier in the literature by Haynes et al^2^; however, instead of a fusiform excision of the skin, a circumferential excision of only the tract was performed and without drain placement. The absence of a drain had no effect on the complication rate.

The fundamental principle of a TCF repair in a burned patient is skin preservation which adds a degree of complexity. In the patient described, a tracheostomy tube remained for more than a year prior to decannulation. The difficulty in closing a persistent stoma in a burned patient is due to the lack of mobility of surrounding tissues from extensive scarring in addition to the lack of subcutaneous tissue that occurs with fascial excision. Moreover, cultured epithelial autograft skin is fragile making dissection of the muscle and skin all the more challenging.

**Figure 3:**
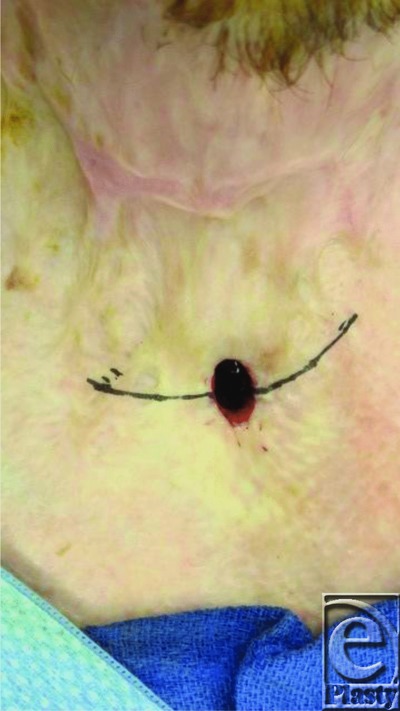
Bilateral linear incisions.

**Figure 4:**
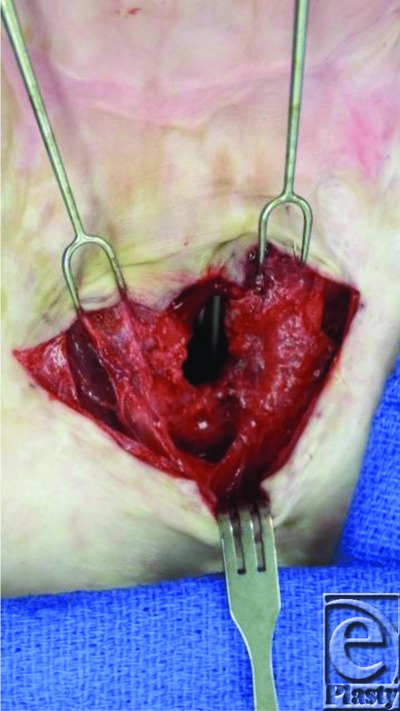
Dissection of sternohyoid muscles.

**Figure 5:**
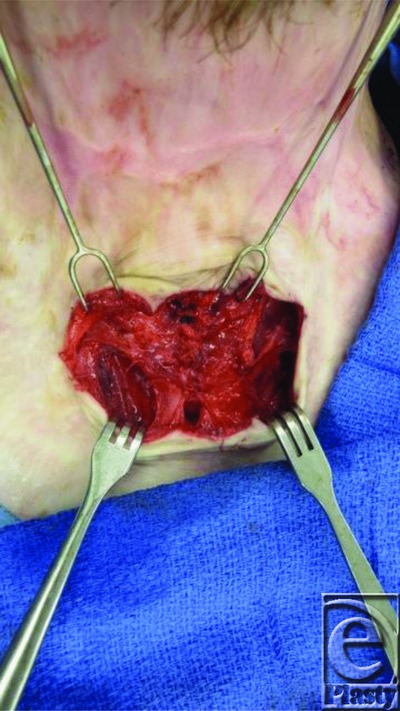
Medial advancement of sternohyoid muscles.

**Figure 6:**
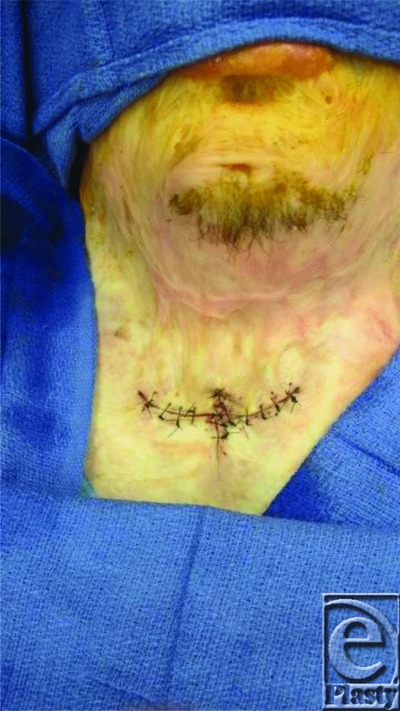
Skin closure.

Bilateral, curvilinear incisions were performed on each side of the stoma allowing us to preserve the skin, dissect, and mobilize the sternohyoid muscles medially (Figs 3 and 4). Because of the significant existing scar tissue, deeper layers of the scar were not closed as in the techniques of Haynes et al and Schroeder et al in order to prevent iatrogenic tracheal stenosis. The sternohyoid muscles were sutured in the midline to occlude the stoma (Fig 5), and the skin was closed transversely in interrupted fashion to provide a 2-layer closure (Fig 6). The patient's fistula has remained closed.
